# Pharmaceutical Processes

**Published:** 1800-03

**Authors:** 


### *Pharmaceutical Processes.*

### *Liquor Anod. Vegetabilis & Naphta Aceti.*

As the acetous æther, and particulary the dulcified spirit of it, have been introduced into medical practice, an improved method of its preparation will not, we trust, be unacceptable to many of our readers.

Dr. Westendorff, of Göttingen,* mixed strong acetous acid with alcohol, having obtained this acid by the decomposition of the acetit of potass with sulphuric acid; and thus, by means of distillation in the usual manner, he prepared the anodyne liquor and the acetous æther; but afterwards the same articles were produced from common acetit of potass. A subsequent attempt was made to prepare the dulcified spirit, and the æther by a compound process, or a double elective attraction; a process the more advantageous, as the separation of the acetic acid, and the production of the æther, and the dulcified spirit, could be effected by a single distillation.

Mr. Voigt, a German apothecary, who first introduced this compound process, found the following method the most satisfactory. Sixteen ounces of acetit of potass † are put into a retort, and through a curved glass funnel, of sufficient length, a mixture of ten ounces of alkohol, and six ounces of sulphuric acid, is gently added; the vessels being well cemented, ten ounces of this mixture are distilled over a slow fire. By mixing the distilled liquor with water, in which some tartarit of potass has been dissolved, it will be found that the greatest part of what has passed over, is the most excellent acetous æther.

As

* Differt. de optima acetum concentratum ejusdemque. Naphtam conficiendi ratione. Göttingæ, 1772.

† For this purpose, the tartarite obtained from the acetit of lead may be employed with equal advantage.

As ſix ounces of ſulphuric acid are inſufficient to ſeparate all the acetous acid, there may ſtill be added to the remainder four ounces of ſulphuric acid and twelve ounces of alkohol, which, when diſtilled, will yield a perfectly dulcified ſpirit of vinegar. In another experiment, Mr. Voigt, from eight ounces and a half of acetit of potaſs, five ounces of ſulphuric acid, and eight ounces and a half of alkohol, obtained nine ounces and ſix drachms of æther, and eleven ounces of dulcified ſpirit of vinegar. This proceſs has alſo been confirmed by Mr. Westrumb, who, from four pounds of cryſtallizable acetit of potaſs, and two pounds of ſulphuric acid, obtained ſeventeen ounces of fuming acetous acid; and ſtill finding part of the ſalt undecompoſed, he poured an additional quantity of eight ounces of ſulphuric acid, and twelve ounces of alkohol, on the remainder, the diſtillation of which afforded ſeven ounces and a half of a dulcified vegetable ſpirit.

Mr. Westrumb, having obſerved that ſome part of the vitriolated tartar, which was produced during the operation, uniformly aſcended in the receiver, and thus frequently interrupted the diſtillation, he prevented this inconvenience by filtering the liquor, after having diſtilled over the firſt portion of the æther.

Mr. Fiedler alſo obtained from the acetit of lead a very good acetous æther. He expoſed eight ounces of the beſt white and cryſtallized acetit of lead, in an earthen veſſel, to ſuch a degree of heat that it no longer ſwelled, when it weighed only ſix ounces and ſeven drachms. This ſugar of lead, in a dry and finely powdered ſtate, he placed into a warm tubulated retort, depoſited this in ſand, joined to it a dry and cloſely fitted receiver, and gradually poured on it a mixture of two ounces of concentrated ſulphuric acid, and three ounces of highly rectified ſpirits of wine. Having well fixed the ſtopper in the retort, he expoſed it to a moderate fire, which he increaſed towards the termination of the proceſs, in order to diſtill the whole over to dryneſs. Of this diſtilled liquor, which had a pleaſant but ſomewhat acid ſmell, two ounces and a half were again diſtilled over, in a ſmall retort, by a very gentle heat. On adding four ounces of lime water, in which ſome alkali had been previouſly diſſolved, the liquor became turbid, and on the ſurface appeared two ounces and ſix drachms of æther, which was removed by a ſmall glaſs ſyringe, or ſyphon. To the reſiduum were added two ounces of ſpirits of wine, which by diſtillation alſo yielded a very pleaſant and ſweet vegetable ſpirit. The ſugar of lead, as well as the ſulphuric acid, not always poſſeſſing the ſame degree of ſtrength, it is neceſſary to aſcertain firſt their due proportion.

Mr.

Mr. Voigt has, for ſeveral years paſt, prepared an excellent vinegar, in no reſpect inferior to that made of wine, by expoſing to a new fermentation the grains remaining from the diſtillation of malt ſpirits. From two gallons of this vinegar, he firſt obtained by diſtillation above a pint of a very ſtrong volatile ſpirit, reſembling the acetous æther. After diſtilling this ſpirit a ſecond time over a gentle ſand bath, he obtained a conſiderable quantity of good acetous æther, together with a pleaſant and dulcified ſpirit of vinegar, of equal quality.

Although Mr. Dollfuss employed acetous acid, prepared from the acetit of lead with ſulphuric acid, he obtained no æther; and ſeems, therefore, to be of Scheele's opinion, that no acetous æther can be produced without the addition of a mineral acid. This aſſertion, however, militates againſt the experiments of Mr. Voigt, and Mr. Lowitz*, who both obtained æther from vinegar alone. Mr. Remler † has likewiſe proved the contrary by deciſive experiments. On decompoſing acetit of potaſs by ſulphuric acid, and again rectifying it over acetit of lead, he obtained a perfectly pure acetous acid, which was not rendered turbid by a ſolution of terra ponderoſa, and conſequently diveſted of all ſulphuric acid. Equal parts of ſuch vinegar and ſpirits of wine yielded excellent acetous æther, and the experiments of Pelletier tend to confirm the ſame fact. *Göttling's Practiſche Vortheile.* Ie Samml. p. 209.

---

### *Teſt of Arſenic being contained in Sulphur.*

In order to aſcertain whether ſulphur contain arſenic, Dr. Richter recommends the following method, as being well adapted for the purpoſe. The ſulphur ought to be firſt decrepitated with four or five times its weight of depurated nitre; the reſiduum diſſolved in diſtilled water; and, the ſolution being filtered, ſome water ſaturated with ſulphat of ſilver muſt be added. If arſenic ſhould be combined with the ſulphur, a browniſh red precipitate will be formed, which is properly arſenicat of ſilver. *Berlin. Jahrbuch der Pharmacie*, 1799. p. 119.

---

### *Method of preparing Æthiops Mineral.*

To obtain Æthiops mineral in the moſt advantageous manner, M. de Roover repeatedly affuſed freſh water on iron filings, either in a freſh or oxydated ſtate, delaying every ſucceſſive affuſion till the preceding had been entirely abſorbed; the

---

the whole was then decanted, minutely triturated with the addition of water, and the fine black precipitate which is thus obtained in conſiderable quantity, was afterwards ſeparated from the water, and dried. This precipitate is of a velvet black colour, and poſſeſſes all the properties of the fineſt Æthiops mineral. *Ibid.* p. 120.

---

### *Medicinal Phosphorus.*

With a view of adapting phoſphorus to medical uſe, Cit. LEROI propoſes to melt it in hot water; to reduce it to a powder, by conſtantly ſhaking it till its ſolidity be reſtored; and to triturate this powder, after diveſting it of humidity, with the addition of oil and ſugar, or the yolk of an egg. *Ibid.* p. 121.

---

### *Mercurial Soap.*

M. MUSSIN PUSHKIN has contrived the following method of producing a mercurial ſoap perfectly ſoluble. He firſt diſſolves ſoap in ſoft water, and combines this with a neutral ſolution of mercury, made in the nitric acid: the precipitate thus formed muſt be properly edulcorated, and afterwards mixed with two or three parts of cauſtic ammonia. By this combination, the mercurial ſoap is rendered completely ſoluble in water. *Ibid.*

