# Errata

**Published:** 1800-04

**Authors:** 


					r. 209, in me pai^rapn> ana in next page, i. v} lor /r-unops mmtraif rew
<?thic>ps Martial: s?

				

